# Data-Driven identification of chemopreventive agents for breast cancer

**DOI:** 10.3906/sag-2003-138

**Published:** 2020-11-03

**Authors:** Deniz Can GÜVEN, Cemal ORHAN, Kazim ŞAHİN, Fatih M. UÇKUN

**Affiliations:** 1 Department of Medical Oncology, Faculty of Medicine, University of Hacettepe, Ankara Turkey; 2 Department of Animal Nutrition, Faculty of Veterinary Medicine, Fırat University, Elazığ Turkey; 3 Division of Hematology-Oncology, Department of Pediatrics and Developmental Therapeutics Program, Norris Comprehensive Cancer Center, University of Southern California Keck School of Medicine (USC KSOM), Los Angeles, CA USA; 4 Ares Pharmaceuticals, St. Paul, MN USA

**Keywords:** Breast cancer, WHI-P131, LFM-A13, stampidine

## Abstract

Preclinical animal models of breast cancer provide the opportunity to identify chemopreventive drugs with single-agent activity as well as effective multi-modality regimens for primary as well as secondary prevention in high-risk persons. Our group has used the 7,12-dimethylbenz(a)anthracene (DMBA) mouse model of carcinogen-induced breast cancer to explore the clinical potential of two tyrosine kinase inhibitors and a nucleoside analog as chemopreventive agents. All three agents exhibited promising preclinical activity both as monotherapy and as components of combination therapy with the standard chemotherapy drug paclitaxel. The tumors developing despite chemoprevention were not only small and grew slowly, but they also displayed a uniquely more pro-apoptotic protein expression profile. Hence, our experimental chemopreventive drugs were capable of preventing the development of aggressive mammary gland tumors with an apoptosis-resistant protein expression profile.

## 1. Introduction

Over 1.5 million women are diagnosed with and over 0.5 million women die of breast cancer each year. Although breast cancer mortality has declined over the past two decades, breast cancer remains the leading cause of cancer deaths in women aged 20 to 59 years [1, 2]. The adverse impact of breast cancer on quality of life, productivity, and survival, as well as health care costs, have prompted intensive research efforts aimed at the identification of breast cancer prevention methods [3]. Effective chemoprevention strategies with selective estrogen receptor modulators (SERMs), such as tamoxifen, and aromatase inhibitors (AIs), including steroidal inhibitors such as exemestane, and nonsteroidal inhibitors, such as anastrozole and letrozole, have been developed to decrease the risk of both invasive and noninvasive breast cancer [3, 4]. An analysis of 83.399 women at high risk for breast cancer who received SERMs for primary chemoprevention showed a 38% reduction in breast cancer incidence [5]. AIs administered as adjuvant therapy after breast surgery were highly effective in reducing the incidence of breast cancer recurrence in women who are diagnosed with breast cancer [6]. In a randomized exemestane study designed to detect a 65% relative decline in invasive breast cancer, 4560 eligible postmenopausal women aged 35 or over had at least one of the following risk factors: *60 years or older; Gail 5-year risk score is more than 1.66% (chances in 100 of invasive breastcancer developing within 5 years); prior atypical ductal or lobular hyperplasia or lobular carcinoma in situ; or ductal carcinoma in situ with mastectomy* , Exemestane decreased the incidence of invasive breast cancers [7]. Available chemoprevention strategies are also related to significant short- and long-term side effects. Guidelines have been developed by the US Preventive Services Task Force (USPSTF) [8, 9] and the American Society of Clinical Oncology (ASCO)[10] regarding the rationale and data-driven, evidence-based use of endocrine therapy for women at high risk for breast cancer. Precision medicines, as well as targeted biotherapeutic agents such as Trastuzumab (Herceptin), offer the opportunity for patient-tailored secondary prevention of breast cancer [11,12]. The discovery of additional effective chemopreventive strategies using chemotherapy drugs, precision medicines, biologics, and natural compounds is a major area of translational research emphasis in contemporary oncology [13]. 

Our recent drug discovery efforts have focused on a data-driven strategy to identify active chemopreventive agents for breast cancer by leveraging an *in vivo* animal model for carcinogen-induced breast cancer. The purpose of the current review is to summarize the salient features of our studies as well as discuss the insights and lessons learned from biomarker analyses. 

## 2. The 7,12-dimethylbenz(a)anthracene model of breast cancer

Mice treated with the chemical carcinogen 7,12-dimethylbenz(a)anthracene (DMBA) develop mammary gland tumors that morphologically resemble human myoepithelial carcinomas and myoepitheliomas [14]. Adenosquamous carcinomas and ductal carcinomas have also been reported in DMBA-treated mice [15].

**Figure 1:**
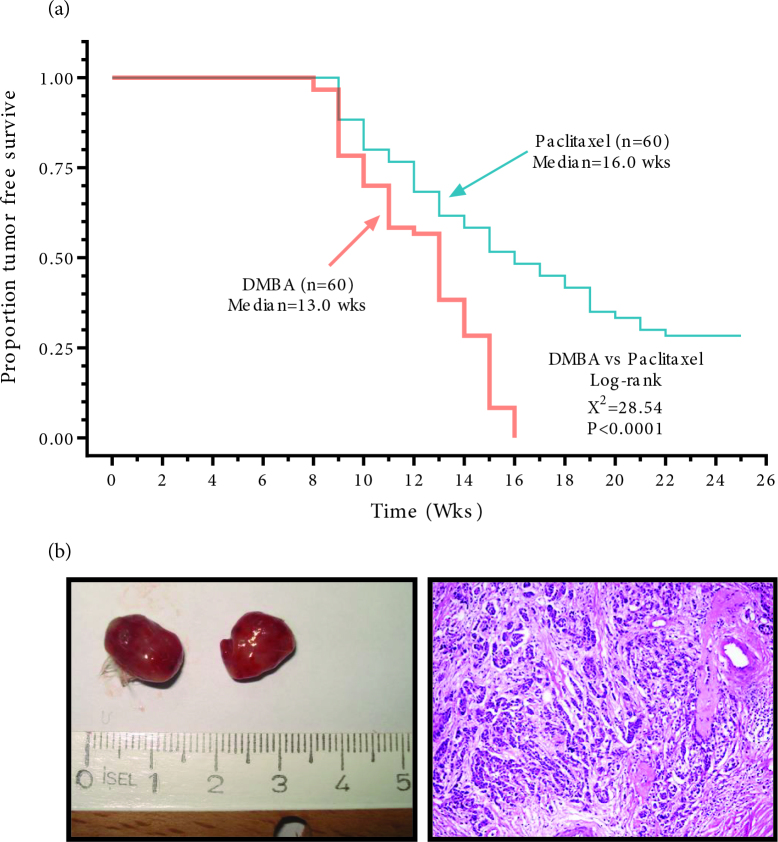
Effects of paclitaxel on the proportion of tumor-free survival (a) and macroscopic appearance and histopathological features (b; H&E X200) of DMBA-induced tumors in the mammary glands of mice.

The polycyclic aromatic hydrocarbons like DMBA activate the aryl hydrocarbon receptor (AhR), which activates a signal transduction cascade that leads to the formation of the mutagenic epoxide of DMBA via cytochromes P450 enzymes. DNA damage due to this mutagenic epoxide is the proposed driving mechanism of malignant transformation in breast tissue [16]. The DMBA initiates and affects multiple steps of carcinogenesis, starting from the inhibition of differentiation in the early stages and followed by cell cycle disruptions such as upregulation of Cyclin D1 and c-Myc, possibly via nuclear factor-κB and Wnt pathways [17]. Disruptions of intracellular members of cell cycle regulation such as PI3K/mTOR/AKT and PTEN were also seen later in the disease course similar to luminal type human breast cancers [18]. In a series of studies, we have used the DMBA model of breast cancer to evaluate the chemopreventive activity of promising new agents in side by side comparison with the standard antibreast cancer drug paclitaxel. All control mice treated with DMBA developed mammary gland tumors within 16 weeks. The median time for progression-free survival (in other terms time to tumor development) was 13 weeks for these mice. The standard breast cancer drug paclitaxel preventedor slowed down the development of mammary tumors significantly and more than 25% of rodents remained tumor-free even after 23 weeks. The progression-free survival was increased to 16 weeks in the paclitaxel arm also (Figure 1).

## 3. Antitumor activity of novel WHI-P131, LFM-A13 and stampidine molecules in DMBA models of breast cancer

**Figure 2:**
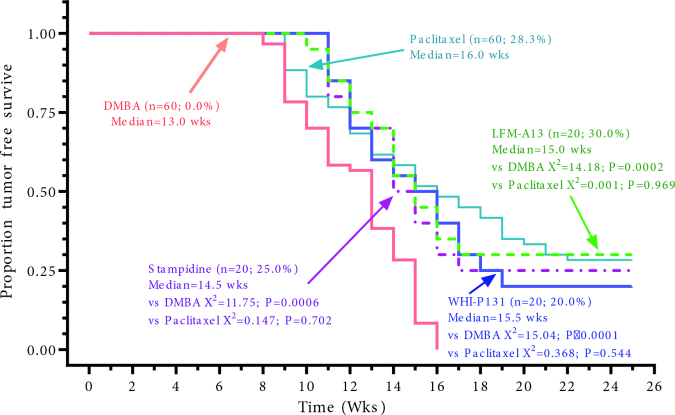
Effects of chemoprevention with paclitaxel, LFM-A13, WHI-P131 and stampidine on proportion tumorfree survive in DMBA-challenged mice.

**Figure 3:**
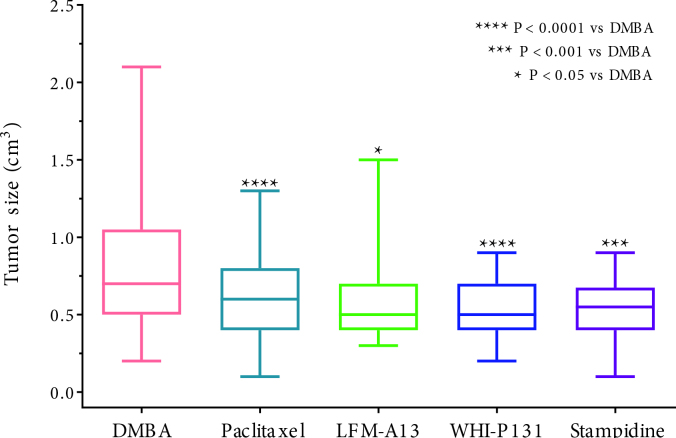
Effects of chemoprevention with paclitaxel, LFM-A13, WHI-P131 and stampidine on the tumor size in DMBA-challenged mice.

**Figure 4:**
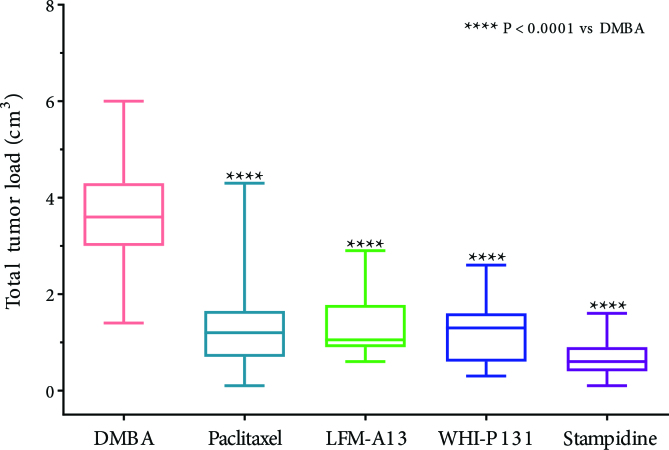
Effects of chemoprevention with paclitaxel, LFM-A13, WHI-P131 and stampidine on total tumor load in DMBA-challenged mice.

In preclinical models, the phosphoramide derivatives of nucleoside analogs showed anti-tumor activities and emerged as potential anticancer drugs [19]. Stampidine is among these agents and it’s an aryl phosphate derivative of stavudine. Its clinical activity was first tested as an anti-HIV agent in preclinical models and also in a phase 1 study[20]. The drug was well-tolerated without dose-limiting toxicities [20]. Stampidine has been shown to act as an epigenetic regulator of gene expression by methylating and silencing the several oncogenic transcription factors[21]. We tested the chemopreventive activity of stampidine in the DMBA model. When co-administered with DMBA, stampidine decreased the number and size of mammary gland tumors that have developed after the DMBA challenge. In addition, the combination of stampidine and paclitaxel synergistically reduced the tumor burden. Another hypothesis-generating point from our study was the demonstration of higher proapoptotic and lower antiapoptotic protein expression in tumors developing in stampidine-treated mice [22]. These results demonstrated that stampidine prevents the development of aggressive breast tumors DMBA challenge. The chemopreventive potency of stampidine was similar to that of paclitaxel as well as two potent kinase inhibitors, WHI-P131 (an inhibitor of HER, Src, and JAK family kinases) [22–25] and LFM-A13 (an inhibitor of TEC and PLK family kinases) [26] (Figures 2–4). These findings illustrate the potential of stampidine as a chemopreventive agent against breast cancer. 

In regards to the two lead kinase inhibitors we tested, WHI-P131 was active in the DMBA induced cancer model. The median tumor free-survival was 18 weeks with WHI-P131 with 20% of mice were tumor in the 18th week of DMBA treatment. This tumor-free survival was significantly improved compared to the control arm (P £ 0.001). The antitumor efficacy was similar to that of paclitaxel (P =0.544) (Figures 2 and 3) [25]. We think that the combination of WHI-P131 with conventional chemotherapeutics can further improve the antitumor efficacy of WHI-P131 as a chemopreventive agent.

The other novel agent we evaluated was LFM-A13 (19). In the DMBA model, LFM-A13 significantly increased the survival to 40% compared to 15% in the control arm. The combination of paclitaxel and LFM-A13 further increased the survival rate to 50% in the 25th week. The tumor incidence and also tumor burden were significantly reduced in the LFM-A13 group [26]. LFM-A13 prolonged the tumor-free interval by 2 weeks (15 vs 13 weeks, P = 0.0002) compared to the control arm. 30% of mice treated with LFM-A13 were tumor-free at 20th week demonstrating long-lasting chemoprevention with the agent. The antitumor efficacy was similar to that of paclitaxel and WHI-P131 (Figures 2–4).

Notably, the tumors developing despite chemoprevention with the kinase inhibitors or stampidine were not only small and grew slowly, but they also displayed a uniquely more proapoptotic protein expression profile. Hence, our experimental chemoprentive drugs are capable of preventing the development of aggressive mammary gland tumors with an apoptosis-resistant protein expression profile. 

## 4. Future perspectives

Although the use of tamoxifen and AI’s for secondary prevention have significantly reduced the risk of are recurrence in hormone receptor-positive breast cancer after standard first-line therapy (5, 27), many high-risk patient populations (especially those with hormone receptor-negative breast cancer) experience a recurrence. Optimizing secondary prevention with drugs used in the adjuvant and prolonged maintenance therapy settings may contribute to a clinically meaningful improvement of survival outcome. Our data in the DMBA model suggest that stampidine, WHI-P131, and LFM-A13 may be useful as chemopreventive agents and as components for secondary chemoprevention protocols. In the DMBA model, the efficacy of these agents in chemoprevention was similar to that of paclitaxel which is among the most effective breast cancer therapies [27]. We are planning to evaluate their activity in combination with tamoxifen and AIs for secondary prevention considering the potential to inhibit different steps of oncogenesis due to different mechanisms of antitumor action. The enhanced potency observed when these agents were combined with paclitaxel is also deserving of further study [22, 25]. LFM-A13 and WHI-P131 were also tested for activity in MMTV/Neu transgenic HER-2 positive models of breast cancer [23, 28], so their synergism with trastuzumab is another area that warrants further research.

As mentioned earlier, the tumors developing despite chemoprevention with the kinase inhibitors or stampidine displayed a pro-apoptotic protein expression profile, which was different from the protein expression profile of aggressive tumors developing in DMBA-treated control mice [22]. Hence, our experimental chemopreventive drugs are capable of preventing the development of aggressive mammary gland tumors with an apoptosis-resistant protein expression profile. It is our working hypothesis that recurrent tumors emerging after chemoprevention with these chemopreventive drugs will, therefore, be substantially more sensitive to available salvage chemotherapy regimens in standard second-line settings. This should contribute to an improved treatment and survival outcome after recurrence. 

One of the most important properties of these rationally designed agents is their tolerability which increases their potential to be used in the combinations. All three agents were well tolerated without dose-limiting hematologic, renal and hepatic toxicities in multiple animal studies [26, 28, 29]. Stampidine did not cause any dose-limiting toxicities in a phase 1 study of thirty HIV patients [20]. However, LFM-A13 and WHI-P131have not been evaluated in human subjects and all three drugs remain to be evaluated to breast cancer patients. 

In summary, stampidine, LFM-A13, and WHI-P131 showed potent chemopreventive activity by reducing the development and number of breast tumors and improving the tumor-free survival outcome in breast cancer animal models. Further models and studies testing the synergism of these agents with another chemopreventive agent like tamoxifen and AI’s and also the use of these agents for primary chemoprevention in women at high risk for breast cancer would seem warranted. 
